# Leader humility and employee proactivity: sequential affective mediation and the moderating effect of role breadth self-efficacy

**DOI:** 10.3389/fpsyg.2025.1592148

**Published:** 2025-09-10

**Authors:** Yanhong Chen, Jingshi Zhao, Meifen Jin, Xiaoyun Lan

**Affiliations:** ^1^College of Business Administration, Huaqiao University, Quanzhou, Fujian, China; ^2^College of Computing, City University of Hong Kong, Hong Kong, China; ^3^College of Business Administration, Zhongnan University of Economics and Law, Wuhan, Hubei, China; ^4^College of Management, Shanghai University, Shanghai, China

**Keywords:** leader humility, positive mood, affective commitment, role breadth self-efficacy, proactive behavior

## Abstract

Despite established links between leader humility and employee proactive behavior, the affective transmission mechanisms and boundary conditions remain theoretically underdeveloped. Guided by Affective Events Theory, this study examines how and when leader humility influences employee proactive behavior through sequential mediation in Chinese organizations. In Study 1, a scenario-based experiment with 105 participants, demonstrates that leader humility enhances employee proactive behavior by fostering positive mood and affective commitment. In Study 2, which collected multi-source survey data from 51 supervisors and 290 subordinates, confirms this chain mediation and further reveals that role breadth self-efficacy amplifies both the effect of affective commitment on proactive behavior and the overall indirect effect of leader humility. Theoretical and practical implications are discussed, along with the directions for future research.

## Introduction

Proactive behavior refers to taking initiative to improve circumstances or create new ones by challenging the status quo rather than passively adapting (Crant, 2000). In today’s turbulent and complex environments, organizations rely heavily on employee proactivity to identify emerging opportunities and mitigate risks (Parker et al., 2010). Not only does it generate substantial organizational benefits, but it is also critical for sustaining long-term organizational development. However, employee proactive behavior remains relatively rare in organizations (Morrison and Phelps, 1999; Parker et al., 2010), largely due to its inherent uncertainty, potential to challenge authority, and tendency to disrupt the stability organizations seek to maintain. These factors often lead employees to opt for silence or passive acceptance, even when they are inclined to act, as they are deterred by the risks of taking initiative (Parker and Collins, 2010). Identifying the factors that motivate employees to engage in proactive behavior is therefore of significant importance (Parker et al., 2010).

Leadership is recognized as a critical factor in shaping employees’ motivation to engage in proactive behavior (Parker et al., 2010). Previous studies have found that some traditional top-down leadership styles, such as transformational leadership (Schmitt et al., 2016), self-sacrificial leadership (Li et al., 2016), and paternalistic leadership (Zhang et al., 2015), have positive effects on employee proactive behavior. However, in dynamic and uncertain organizational environments, traditional top-down leadership that overemphasizes the leader’s authority and influence is insufficient; instead, there is a growing call for bottom-up approaches that highlight employees’ influence in the leadership process (Asghar et al., 2022; Hu et al., 2018; Owens and Hekman, 2012). Leader humility, characterized by approachability, accurate self-awareness, an appreciation of others’ strengths and contributions, and openness to feedback (Chen et al., 2017), typically expresses significant regard for employees and acknowledges their efforts (D'Errico, 2019). Such behavioral displays are often seen as reflecting sincere positive emotions (Feng et al., 2024), which is critical for employees when confronting the “proactivity dilemma” (Parker et al., 2010). Recent studies have linked leader humility to proactive behavior through mechanisms like psychological empowerment (Chen et al., 2018; El-Gazar et al., 2022), moral self-efficacy (Owens et al., 2019), and need satisfaction (Chen et al., 2021). They have largely overlooked its impact on employees’ affective reactions, however, which hinders the theoretical advancement of leader humility research (Wang et al., 2018). Given that affective responses are a core psychological mechanism driving work outcomes, examining employees’ emotional reactions is essential to fully understanding the effectiveness of leader humility.

Toward this end, this study draws on Affective Events Theory (AET) (Weiss and Cropanzano, 1996), which emphasizes how affect-laden events shape employees’ emotional responses, attitudes, and subsequent behaviors. Within leadership research, AET applications highlight that leader behaviors function as key affective events, eliciting diverse emotional reactions from subordinates and thereby influencing their behavioral outcomes (Cropanzano et al., 2017). This framework is particularly relevant in Chinese Confucian-influenced contexts, where supervisor–subordinate interactions are characterized by strong affective underpinnings (Farh et al., 1998). In such contexts, employees’ emotional experiences are deeply tied to leader behaviors. Humble leader behaviors, for instance, acknowledging subordinates’ contributions and openness to feedback, align with Confucian values of “modesty” and “respect for others,” which makes them more likely to be interpreted as positive affective events. AET thus provides a precise lens to unpack how these culturally congruent behaviors trigger emotional reactions and subsequent proactive behavior. Building on this premise, this study proposes that leader humility acts as an affect-laden event that elicits positive affective reactions (i.e., positive mood) and positive work attitudes (i.e., affective commitment), which, in turn, promote proactive behavior.

While the sequential mediation of positive mood and affective commitment explains how leader humility fosters proactive behavior, AET also posits that individual differences may influence how work events impact employees’ emotional and behavioral responses (Weiss and Cropanzano, 1996). A critical individual difference in this process is role breadth self-efficacy, defined as the confidence in one’s ability to execute a proactive, expanded role that transcends formally prescribed job requirements (Parker, 1998). Proactive behavior, by nature, involves actions that exceed formal job requirements or challenge the status quo. These behaviors are inherently fraught with psychological risk and uncertainty (Parker and Collins, 2010). Because such behavior demands confidence in one’s capacity to navigate ambiguity and perform beyond expectations, this study focuses on employees’ role breadth self-efficacy as the moderator of the above indirect relationship.

Our study makes three contributions to the literature: First, this study advances understanding of the link between leader humility and employee proactive behavior by providing empirical evidence for an affect-based mechanism. While prior research has linked leader humility to proactive behavior (e.g., Chen et al., 2018; Chen et al., 2021), our focus on affective pathways (i.e., positive mood and affective commitment) not only provides a new theoretical perspective for deepening understanding of how leader humility influences employees’ willingness to engage in proactive behavior but also addresses the neglect of emotional processes in existing humility-proactivity research (Kelemen et al., 2023; Wang et al., 2018).

Second, by examining the moderating effect of role-breadth self-efficacy, this study identifies a key boundary condition that shapes when leader humility translates into proactive behavior through affective pathways. This responds to the call to explore individual differences in leader humility research (Kelemen et al., 2023), clarifying that humility’s affective effects are amplified among employees confident in their ability to perform proactive roles.

**Figure 1:**
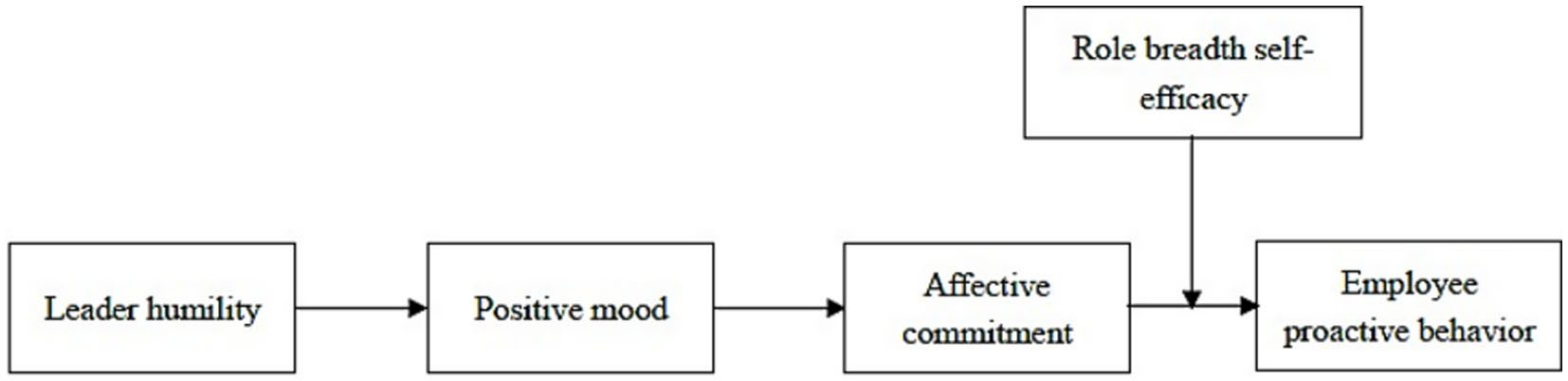
Conceptual model. AET logic: workplace event (leader humility) ® emotion reaction (positive mood) ® attitude (affective commitment) ® behavior (proactive behavior), with individual differences (role breadth self-efficacy) acting as a moderator.

Lastly, this study advances Chinese management literature by illuminating humility as a culturally rooted concept, which has been recognized as a foundational leadership virtue in Chinese contexts (Chen et al., 2017). Our findings reveal that Chinese employees tend to develop emotional ties with leaders who embody this traditional value, valuing their modesty and recognition. Moreover, as Wan et al. (2022) noted, existing research has paid insufficient attention to theorizing and examining the interplay between leadership and affective processes in Chinese organizations. By applying AET, this study addresses the need by emphasizing the role of emotional processes in leadership, demonstrating that Chinese employees perceive humble leader behaviors as positive “affective events” that foster proactive action. The research model is presented in Figure 1.

To test the proposed model, we adopt a dual-study design. Study 1 uses a scenario-based experiment to establish causal relationships in the affective mediation chain. Study 2 employs a multi-source survey to validate the full theoretical model.

## Theoretical background and hypothesis development

The concept of leader humility was initially developed in the United States. Owens and Hekman (2012) categorized its behavioral expressions into three main aspects: admitting personal limitations, faults, and mistakes; highlighting followers’ strengths and contributions; and exemplifying teachability. The majority of research on leader humility conducted in China has adopted this conception and measurement to explore the effects of leader humility (e.g., Lin et al., 2019; Zhang and Liu, 2019). Despite academic conceptualizations of humility originated in the West, it constitutes a fundamental principle within Chinese Confucian cultural traditions. Chiu et al. (2012) posit that humility in the Chinese context may have its uniqueness in the structural dimension. If we directly apply the conception and measurement of leader humility developed in the United States to China, some portions of the domain may be missing (Liden, 2012). Given the cultural embeddedness of leadership constructs, this study adopts Chen et al. (2017) culturally grounded conceptualization of leader humility within Chinese organizational contexts to investigate its influence on employee proactive behaviors. Defined through a behavioral lens, leader humility in this framework comprises four dimensions: “(1) approachability, (2) accurate self-awareness, (3) an appreciation of others’ strengths and contributions, and (4) openness to feedback.”

### Leader humility and positive mood

Positive mood, defined as a state of enthusiasm, activation, and alertness (Watson et al., 1988), represents a key immediate affective reaction within the AET framework (Weiss and Cropanzano, 1996). AET posits that emotional states are elicited through individuals’ interpretation of workplace stimuli. Research on leadership has conceptualized leader behaviors as discrete experiences that shape employees’ affective states (Bader et al., 2023; Cropanzano et al., 2017). Effective leaders, for instance, can elicit employees’ affective states through behaviors such as providing inspiration, offering recognition, and delivering feedback (Dasborough, 2006). In our context, leaders’ humble behaviors, such as recognizing employees’ contributions and being open to their feedback, are likely interpreted by employees as positive workplace events, thereby influencing their positive mood.

Specifically, by identifying and appreciating employees’ strengths, efforts, and contributions, humble leaders help employees perceive that leaders believe in them and value their abilities, thereby triggering feelings of enthusiasm. By remaining open to employees’ opinions and feedback, humble leaders enhance employees’ sense of self-control and interest in their work, which in turn heightens their emotional experiences of excitement and happiness at work. By treating employees with inclusiveness and respect, humble leaders make employees feel that leaders respond with optimism rather than criticism when problems arise, ultimately boosting employees’ positive mood at work (Ye et al., 2018). Thus, we propose that:

*H1*: Leader humility is positively related to positive mood.

### Leader humility, positive mood, and affective commitment

Certain work events can also result in certain long-term work attitudes through affective reactions (Weiss and Cropanzano, 1996). Weiss and Cropanzano (1996) have noted that these work attitudes comprise both an affective element and a cognitive judgment element. As employees usually associate their leaders with the organization and see them as symbols of the organization (Biron and Bamberger, 2012), employees who receive humble leader behaviors may have positive feelings and evaluative judgments about their jobs and organizations. Affective commitment is such an affect-based bond to the organization, and is defined as “an emotional attachment to, identification with, and involvement in the organization” (Meyer and Allen, 1991, p. 67). Accumulated research has indicated that leaders expressing admirable behavior are likely to enhance employees’ affective commitment (Asghar et al., 2023; Lapointe and Vandenberghe, 2017; Ribeiro et al., 2020). Thus, we propose that leader humility, as a recurring positive affective event, fosters affective commitment.

First, humble leaders are approachable, respectful, and considerate towards their subordinates (Chen et al., 2017), which can meet employees’ spiritual needs at a higher level. Such emotional satisfaction makes employees generate a high sense of belonging, identity, and attachment to the organization, thus showing a high level of affective commitment to the organization. Second, humble leaders accurately see their strengths and limitations by transparent disclosure of personal limits, acknowledging mistakes, and asking for feedback about themselves (Chen et al., 2017; Owens et al., 2013). These behaviors make employees convinced that they are working with a psychologically healthy leader who can make good decisions and take appropriate actions, thereby enhancing the organizational effectiveness (Argandona, 2015). Consequently, employees develop pride in belonging to their organization and develop higher affective commitment. Third, humble leaders acknowledge and admire employees’ strengths and contributions (Chen et al., 2017; Owens et al., 2013). Such humble behaviors signal that employees’ inputs are important and valued, which makes employees feel that they are perceived as trustworthy, significant to, and influential on the work (Cho et al., 2021; Morris et al., 2005). These favorable experiences further promote employees to develop an affective attachment to their organization. Thus, we propose the following hypothesis:

*H2*: Leader humility is positively related to affective commitment.

Positive mood at work may enable employees to become more affectively committed to their organization. As suggested by the broaden-and-build theory (Fredrickson, 2001), employees experiencing a positive mood have broadened cognition and attention, and tend to find positive meaning in ordinary events, which makes employees consider working for their organization as enjoyable and reflect well on their organization. Moreover, positive mood at work makes the job meaningful and intrinsically rewarding, which is a core predictor of affective commitment (Eby et al., 1999). Previous studies also provided some empirical evidence for the positive link between positive mood at work and affective commitment. For example, Lilius et al. (2008) found that the positive mood sparked by compassion is positively related to affective commitment to the organization. Similarly, Rego et al. (2011) study found that employees who experience a higher positive mood develop higher affective commitment. Combining the above arguments, positive mood can act as a bridge that links leader humility with affective commitment. According to AET, leader humility functions as a salient affective event. Employees’ appraisals of these humble behaviors (e.g., approachability, accurate self-awareness, openness to feedback) trigger the immediate affective state of positive mood. This makes positive mood the most direct and proximal emotional pathway through which humble leader behaviors influence employees’ subsequent attitudes. Consistent with the aforementioned “Event-Reaction-Attitude” framework of AET, we propose the following hypothesis:

*H3*: Positive mood mediates the relationship between leader humility and affective commitment.

### Affective commitment and employee proactive behavior

AET posits that work attitudes generated from work events and affective reactions will result in distal judgment-driven behaviors. We propose that affective commitment affected by leader humility and positive mood will promote employees to engage in proactive behavior.

Affective commitment reflects employees’ identification with the organizational objectives and values and a feeling of pride in their organization (Meyer and Allen, 1991). Employees who demonstrate greater affective commitment have a strong desire to remain with their organization. Thus, they are autonomously motivated to exert effort for organizational goals, even when these require actions that go beyond in-role responsibilities. Specifically, employees with high levels of affective commitment have a strong sense of ownership and regard organizational interests as their own. Those employees are more likely to initiate proactive behaviors voluntarily (e.g., sharing creative ideas, improving work methods, and making constructive suggestions) to aid organizational success, even when such behaviors bring problems and challenge the status quo (LePine and Van Dyne, 1998). In line with our reasoning, previous studies found that highly affectively committed employees tend to exhibit more proactive behaviors (Lapointe and Vandenberghe, 2018; Strauss et al., 2009; Wang et al., 2014). Thus, we propose the following hypothesis:

*H4*: Affective commitment is positively related to employee proactive behavior.

### Positive mood and affective commitment as sequential mediators

AET depicts employees’ behaviors as a process that occurs through affective reactions and work attitudes, initiated by exposure to work events and culminating in behavioral outcomes (Weiss and Cropanzano, 1996). Combining the above arguments (Hypotheses 1, 2, 3, and 4), we posit that leader humility may lead employees to experience positive mood and that affective experience at work leads to pleasant affective associations with the organization and accumulates into strengthened affective commitment, and ultimately enables employees to engage in proactive behavior to benefit the organization. Thus, we propose the following hypothesis:

*H5*: Positive mood and affective commitment sequentially mediate the relationship between leader humility and employee proactive behavior.

### Role breadth self-efficacy as a moderator

AET emphasizes that individual differences may potentially influence the effect of work events on employees’ emotional and behavioral reactions (Eissa and Lester, 2017; Weiss and Cropanzano, 1996). In this study, proactive behaviors go beyond job requirements and involve risks like challenging supervisors (Fast et al., 2014). Thus, even with autonomous motivation (affective commitment), employees’ assessment of whether such behaviors will succeed remains critical (Parker et al., 2010). Role breadth self-efficacy refers to “the extent to which people feel confident that they are able to carry out a broader and more proactive role, beyond traditional prescribed job requirements” (Parker, 1998, p. 835). It has been shown to moderate how experiences translate into proactive behavior (Den Hartog and Belschak, 2012); yet, its role in the affective pathways of leader humility remains underexplored.

We posit that employees’ role breadth self-efficacy strengthens the positive effect of affective commitment on proactive behavior. For employees with higher role breadth self-efficacy, their confidence in overcoming obstacles and managing uncertainty enables them to act on their emotional attachment to the organization, making affective commitment a stronger predictor of proactive behavior. Conversely, low role breadth self-efficacy undermines this translation, as employees doubt their capacity to execute proactive roles, even when motivated by affective commitment (Den Hartog and Belschak, 2012). Thus, for employees with lower levels of role breadth self-efficacy, the positive relationship between affective commitment and proactive behavior may be weakened. Accordingly, we propose that:

*H6*: Employees’ role breadth self-efficacy moderates the relationship between affective commitment and proactive behavior, such that the positive relationship becomes stronger when the level of role breadth self-efficacy is higher.

Combining the above-mentioned arguments with our theoretical development for Hypotheses 1, 2, 3, 4, and 5, we further posit that employees’ role breadth self-efficacy would moderate the indirect effects of leader humility on employee proactive behavior via positive mood and affective commitment. Specifically, higher levels of role breadth self-efficacy provide employees the confidence to engage in proactive behavior. With such confidence, positive mood, and affective commitment fueled by leader humility enable employees to engage in more proactive behavior. In contrast, lower levels of role breadth self-efficacy make employees fear that they cannot do extra-role behaviors and cannot get the expected outcomes. Even though they have reasons to do proactive behaviors, lower levels of role breadth self-efficacy limit affectively committed employees’ possibility to act proactive behaviors. Thus, the following hypothesis is proposed:

*H7*: Employees’ role breadth self-efficacy moderates the indirect relationships between leader humility and employee proactive behavior through positive mood and affective commitment, such that the indirect relationships become stronger when the level of role breadth self-efficacy is higher.

**Figure 2:**
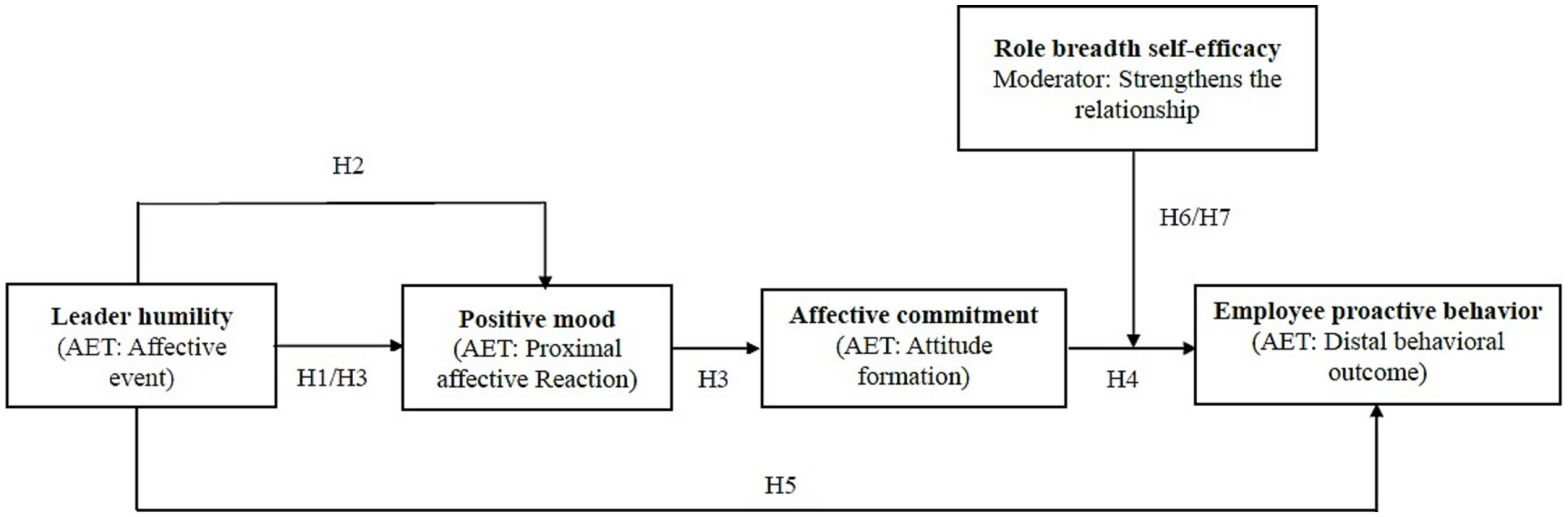
Theoretical model grounded in AET.

To further clarify how AET underpins the proposed relationships, a model depicting the AET-based mechanisms is presented in Figure 2. This model integrates the sequential mediation of positive mood and affective commitment, alongside the moderating role of role breadth self-efficacy, aligning with the core tenets of AET.

## Study 1

### Methods

#### Sample and procedure

The sample comprised 105 MBA students from a university in northern China. We selected this group for two primary reasons: First, MBA students typically possess substantial work and managerial experience, enhancing the study’s realism by increasing both (1) the similarity between the experimental and natural settings and (2) “the subjective experience of being personally immersed in the situation described in the vignette” (Aguinis and Bradley, 2014, p. 11). Second, as MBA programs aim to develop supervisory skills and involve students in performance evaluations (Castilla and Benard, 2010), participants are generally highly motivated to bridge theory and practice. This motivation likely fosters deeper engagement with the tasks, bolstering the validity of our findings. Participants had an average age of 29.53 (SD = 3.58), and 61.9% were female. Following Rego et al. (2019), participants were randomly assigned to one of two conditions: 55 to the humble leader condition and 50 to the transactional leader (control) condition. After reading a scenario describing the assigned leader and imagining working with them, participants completed manipulation checks and then reported their affective commitment, positive mood, and proactive behavior.

#### Leader humility manipulation

The leader scenarios (humble vs. transactional) adapted from Rego et al. (2019) have been validated in recent studies (e.g., Liu et al., 2024; Zettna et al., 2024; Zhu et al., 2019). To further ensure validity for Chinese organizational contexts, we conducted a pre-test with 20 MBA students (with an average of 6.2 years of work experience) who evaluated the scenarios for realism and relevance. Results indicated high perceived realism (M = 4.3/5, SD = 0.62) and relevance to typical leadership behaviors in Chinese firms (M = 4.1/5, SD = 0.58), with no suggestions for major revisions. In the humble leader scenario, the leader was described as respectful, appreciative of followers’ contributions, self-aware of strengths and weaknesses, and open to learning from employees. In the transactional leader scenario, the leader focused on rewarding task completion, punishing unmet expectations, and intervening only in serious problems. Full scenario details are provided in Table A1.

#### Measures

Following Brislin (1980) translation–back translation approach, an English teacher translated the English items into Chinese. Two doctoral students who were fluent in both Chinese and English then translated these Chinese items back into English. At last, they discussed and resolved the discrepancies between the two English versions. Unless indicated otherwise, participants’ responses are made on a 5-point Likert scale ranging from “1 = strongly disagree” to “5 = strongly agree.”

*Affective commitment*. Affective commitment was measured using the 6-item subscale of organizational commitment from Meyer et al. (1993). The sample item was “I feel as if this organization’s problems are my own.” The Cronbach’s alpha estimate for this scale was 0.85.

*Positive mood*. Positive mood was measured using the nine-item scale adapted for the Chinese context by Qiu et al. (2008) from the Positive Affect subscale of the Positive and Negative Affect Schedule (PANAS) developed by Watson et al. (1988). Participants were asked to report the extent to which they felt each affective descriptor (e.g., excited, proud, etc.) (ranging from 1 = not at all to 5 = a great deal) after reading the scenario. The Cronbach’s alpha estimate for this scale was 0.90.

*Proactive behavior*. Proactive behavior was measured using the three-item scale from Griffin and Parker (2007). The sample item was “I initiate better ways of doing my core tasks.” The Cronbach’s alpha estimate for this scale was 0.83.

### Results and discussion

#### Manipulation check

Participants were asked to respond to a one-item manipulation check: “I would characterize Dong Wang as a humble leader” (1 = strongly disagree to 5 = strongly agree). Analysis of the independent sample t-test showed that participants in the leader humility condition rated the leader as significantly humbler (*M* = 4.31, *SD* = 0.54) than participants in the control condition (*M* = 1.38, *SD* = 0.53). The difference was statistically significant (*t* = 28.00, *p* < 0.001), which suggests an effective manipulation of eliciting participants to imagine themselves working with a humble leader.

#### Descriptive statistics

Table 1 shows the means, standard deviations, and Pearson’s bivariate correlations in Study 1.

**Table 1:** Means, standard deviations, and intercorrelations among variables for Study 1.

Variables	Mean	SD	1	2	3	4	
1. Gender^a^	0.06	0.49					
2. Age	29.53	3.58	0.03				
3. Leader humility	2.80	1.62	−0.11	0.08			
4. Positive mood	2.93	0.62	0.03	0.11	0.53^**^		
5. Affective commitment	3.04	0.68	0.01	0.20^*^	0.52^**^	0.50^**^	
6. Proactive behavior	3.73	1.05	0.01	0.05	0.68^**^	0.57^**^	0.56^**^

*N* = 105; ^a^Gender was coded “0” for “male” and “1” for “female”; ^*^p < 0.05; ^**^p < 0.01.

#### Hypothesis testing

**Figure 3:**
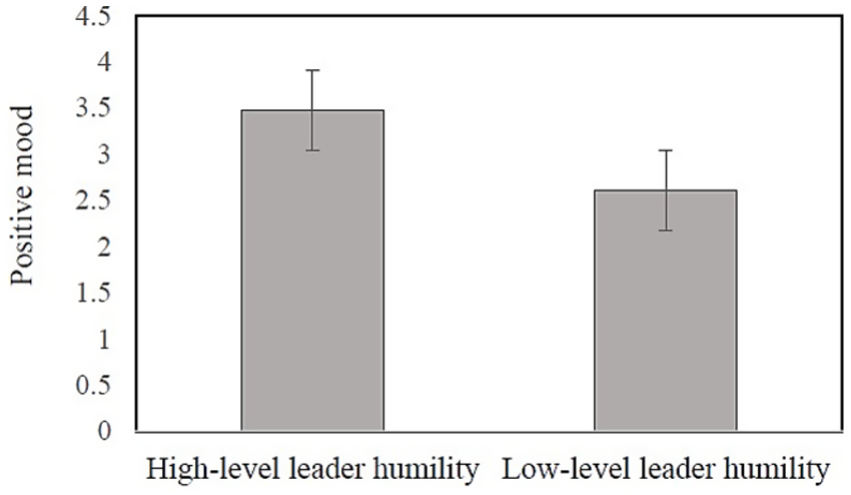
Mean comparison of positive mood (Study 1). Error bars represent standard errors.

**Figure 4:**
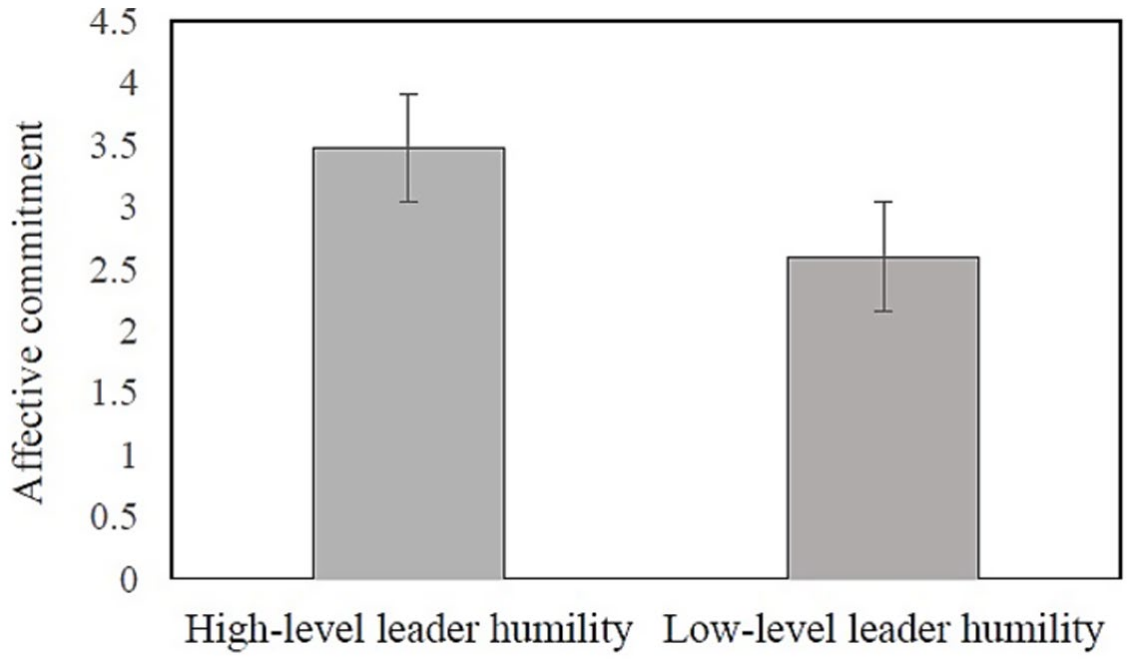
Mean comparison of affective commitment (Study 1). Error bars represent standard errors.

We conducted the independent sample t-tests to compare the mean values of affective commitment, and positive mood in the two conditions of higher levels of leader humility and lower levels of leader humility. As depicted in Figures 3, 4, the participants in the condition of higher levels of leader humility reported a higher degree of positive mood (*M* = 3.29, SD = 0.44) and affective commitment (*M* = 3.47, SD = 0.48) than did those in the condition of lower levels of leader humility (*M* = 2.56, SD = 0.57; *M* = 2.60, SD = 0.65). Their differences were statistically significant (*t* = 7.38, *p* < 0.001; *t* = 7.84, *p* < 0.001, respectively). These results lend support to the causality of leader humility to positive mood and affective commitment.

We followed the recommendations of Hayes (2013) to use Model 6 of the PROCESS macro in SPSS to verify whether positive mood mediates the relationship between leader humility and affective commitment, and positive mood and affective commitment sequentially mediate the relationship between leader humility and proactive behavior. We calculated the bias-corrected bootstrap 95% confidence interval of the indirect effect with 10,000 repetitions. Results showed that leader humility was positively related to employee affective commitment through positive mood (*β* = 0.08, SE = 0.03, 95% CI [0.01, 0.14]), supporting Hypothesis 3; leader humility had a serial indirect effect on proactive behavior through positive mood and affective commitment (*β* = 0.12, SE = 0.05, 95% CI = [0.01, 0.03]), supporting H5.

### Discussion

The findings demonstrate that leader humility is positively related to positive mood and affective commitment. Although this scenario study can support the causal direction of our hypotheses, it lacks an actual organizational context, limiting the external validity. In addition, this study did not test our moderating hypotheses. To address these limitations, we conducted a multi-source field survey (Study 2) to improve the external validity of the first study in testing Hypotheses 1, 2, 3, 4, and 5, and examine all proposed relationships.

## Study 2

### Methods

#### Participants and procedure

The sample consisted of full-time employees from diverse enterprises in northeastern China, including four manufacturing enterprises, three service enterprises, and two financial enterprises. To mitigate common method variance and social desirability biases, a two-source data collection approach was employed (supervisors and subordinates). The research team coordinated with senior management at each organization to secure approval. Before distributing the questionnaires, we explained the research objectives to the participants, emphasizing that the survey was for academic purposes only and ensuring full anonymity and voluntary participation. With support from the human resources department, paper-and-pencil surveys were distributed to 60 supervisors and 355 subordinates.

Final response rates reached 85% for supervisors (*n* = 51) and 81.69% for subordinates (*n* = 290), yielding an average of 5.68 subordinates per supervisor (range: 3–10). Supervisor sample included 80.39% male respondents, a mean age of 41.78 years (SD = 7.33), 19.08 years of organizational tenure (SD = 8.32), and 90.19% holding bachelor’s degrees. Subordinate participants comprised 61% male employees, with a mean age of 38.94 years (SD = 9.09), 15.08 years of tenure (SD = 10.57), and 82.10% holding bachelor’s degrees.

#### Measures

As in Study 1, we adopted the translation–back translation approach to translating from English to Chinese. Unless indicated otherwise, participants’ responses are also made on a 5-point Likert scale ranging from “1 = strongly disagree” to “5 = strongly agree.” Affective commitment, positive mood, and proactive behavior were measured with the same scales that were used in Study 1. The difference is that supervisors reported how frequently their subordinates acted proactive behavior. The Cronbach’s alpha estimates for these scales were 0.90, 0.91, and 0.80, respectively.

##### Leader humility

Subordinates rated leader humility with 14 items developed by Chen et al. (2017) in the Chinese context. The sample item was “My supervisor is full of affability and I feel very relaxed with him/her.” The Cronbach’s alpha estimate for this scale was 0.95.

##### Role breadth self-efficacy

Role breadth self-efficacy was measured using the 7-item scale from Parker et al. (2006). The sample item was “I am confident to present information to a group of colleagues.” The Cronbach’s alpha estimate for this scale was 0.92.

*Control* var*iables*. We controlled for employees’ age, gender, education, and organizational tenure because previous studies have indicated that these variables may have effects on proactive behavior (Burnett et al., 2015; Grant et al., 2009; Shin and Kim, 2015). Moreover, following prior studies (e.g., Wu and Parker, 2017; Wu et al., 2018), we also controlled for subordinates’ proactive personalities because it has been identified as a key individual difference to affect employee proactive behavior (Parker et al., 2006).

#### Analytic strategy

As proactive behavior ratings were nested within the supervisor data, we conducted a one-way ANOVA analysis to examine whether proactive behavior ratings varied across different supervisors (Bliese, 2000). Results show a non-significant effect of supervisors on proactive behavior ratings [*F*(28, 114) = 6.13, *p* > 0.05; ICC1 = 0.01]. These results indicate that there is minimal nesting effect. We thus tested the proposed hypotheses with multiple moderated regressions at the individual level rather than hierarchical linear modeling.

### Results

#### Confirmatory factor analysis

We conducted a confirmatory factor analysis to test the discriminant validity of the study variables using Mplus 7. Considering the large number of items and the relatively small sample size, parcels of items were created as recommended by Little et al. (2002). By adopting the item-to-construct balanced approach, we created three parcels for each of the unidimensional constructs that measured by over three items (i.e., positive mood, affective commitment, and role breadth self-efficacy). For leader humility, a dimensional construct, we adopt the internal-consistency approach to create four parcels to represent its four facets. Results showed that the hypothesized five-factor model provided a better fit to the data (χ^2^ = 168.80, *df* = 94, CFI = 0.98, TLI = 0.98, RMSEA = 0.05) than any other alternative models (e.g., a four-factor model that combined positive mood and affective commitment: χ^2^ = 580.38, *df* = 98, CFI = 0.88, TLI = 0.86, RMSEA = 0.13; a three-factor model that combined positive mood, affective commitment, and role breadth self-efficacy: χ^2^ = 1235.86, *df* = 101, CFI = 0.72, TLI = 0.67, RMSEA = 0.20; a two-factor model that combined positive mood, affective commitment, role breadth self-efficacy, and proactive behavior: χ^2^ = 1402.98, *df* = 103, CFI = 0.68, TLI = 0.63, RMSEA = 0.21; and a one-factor model that combined all variables: χ^2^ = 1571.05, *df* = 104, CFI = 0.64, TLI = 0.58, RMSEA = 0.22).

Although we adopted the multi-source measurement design, there may be a problem of common method variance (CMV). Following the recommendation from Podsakoff et al. (2003), we added an unmeasured latent method factor to the measurement model to test CMV. The model with the CMV factor showed (*χ^2^* = 185.62, *df* = 92, CFI = 0.98, TLI = 0.97, RMSEA = 0.05). Compared to the fit of the five-factor model, the fit indicators of the model with the CMV factor do not vary by more than 0.01, which shows that CMV is not a serious problem in our study.

#### Descriptive statistics

Table 2 shows the means, standard deviations, and Pearson’s bivariate correlations in Study 2.

**Table 2:** Means, standard deviations, and intercorrelations among variables for Study 2.

Variables	Mean	SD	1	2	3	4	5	6	7	8	9	10
1. Gender^a^	0.39	0.49										
2. Age	38.94	9.09	0.01									
3. Education^b^	2.83	0.51	0.04	−0.41^**^								
4. Organizational tenure	15.08	10.57	0.04	0.93^**^	−0.44^**^							
5. Proactive personality	3.67	0.50	−0.06	0.14^*^	−0.05	0.12^*^	(0.85)					
6. Leader humility	3.88	0.75	−0.09	−0.08	−0.04	−0.08	0.26^**^	(0.97)				
7. Positive mood	3.11	0.68	−0.06	0.05	−0.09	0.04	0.42^**^	0.49^**^	(0.91)			
8. Affective commitment	3.91	0.72	−0.01	0.23^**^	−0.18^**^	0.22^**^	0.43^**^	0.42^**^	0.56^**^	(0.90)		
9. Role breadth self-efficacy	3.74	0.79	−0.11	−0.06	−0.04	−0.07	0.37^**^	0.65^**^	0.51^**^	0.43^**^	(0.95)	
10. Proactive behavior	3.99	0.66	−0.06	0.15^*^	−0.10	0.14^*^	0.32^**^	0.29^**^	0.49^**^	0.63^**^	0.33^**^	(0.74)

*N* = 290 subordinates, 51 supervisors; ^*^p < 0.05, ^**^p < 0.01, ^***^p < 0.001 (two-tailed); Age and organizational tenure were measured in years; ^a^Gender was coded “0” for “male” and “1” for “female”; ^b^Education was coded “1” for “lower bachelor’s degree,” “2” for “bachelor’s degree,” and “3” for “master’s degree or higher.” Cronbach’s alphas are reported in parentheses along the diagonal; SD = standard deviation.

#### Hypothesis testing

We first ran OLS regression using SPSS to test Hypotheses 1, 2, and 4. Results are shown in Table 3. Model 2 in Table 3 shows that leader humility was significantly and positively related to positive mood (*β* = 0.37, SE = 0.05, *p* < 0.001). Model 5 in Table 3 shows that leader humility was significantly and positively related to affective commitment (*β* = 0.34, SE = 0.05, *p* < 0.001). Model 7 in Table 3 shows that affective commitment was significantly and positively related to proactive behavior (*β* = 0.55, SE = 0.05, *p* < 0.001). Thus, Hypotheses 1, 2, and 4 received support.

**Table 3:** Hierarchical regression results for Study 2.

Variables	Positive mood		Affective commitment			Proactive behavior			
	Model 1	Model 2	Model 3	Model 4	Model 5	Model 6	Model 7	Model 8	Model 9
Intercept	1.39^**^(0.46)	0.20 (0.44)	1.74^***^ (0.48)	1.07^*^(0.43)	0.64 (0.47)	2.50^***^ (0.46)	1.55^***^(0.39)	1.46^***^(0.40)	1.43^***^ (0.39)
Gender	−0.04 (0.08)	−0.00 (0.07)	0.02 (0.08)	0.04 (0.07)	0.06 (0.07)	−0.06 (0.08)	−0.07 (0.06)	−0.06 (0.06)	−0.07 (0.06)
Age	0.00 (0.01)	0.00 (0.01)	0.01 (0.01)	0.01 (0.01)	0.01 (0.01)	0.01 (0.01)	−0.00 (0.01)	−0.00 (0.01)	−0.00 (0.01)
Organizational tenure	−0.00 (0.01)	−0.00 (0.01)	0.00 (0.01)	0.00 (0.01)	0.00 (0.01)	0.00 (0.01)	0.00 (0.01)	0.00 (0.01)	0.00 (0.01)
Education	−0.12 (0.08)	−0.07 (0.07)	−0.14 (0.08)	−0.08 (0.07)	−0.09 (0.08)	−0.06 (0.08)	0.02 (0.07)	0.02 (0.07)	0.02 (0.07)
Proactive personality	0.57^***^(0.07)	0.42^***^(0.07)	0.59^***^(0.08)	0.32^***^(0.07)	0.45^***^ (0.07)	0.40^***^(0.07)	0.08 (0.07)	0.06 (0.07)	0.04 (0.07)
Leader humility		0.37^***^(0.05)			0.34^***^ (0.05)				
Positive mood				0.48^***^(0.06)					
Affective commitment							0.55^***^ (0.05)	0.53^***^ (0.05)	0.55^***^ (0.05)
Role breadth self-efficacy								0.06 (0.05)	0.06 (0.05)
Affective commitment × Role breadth self-efficacy									0.13^**^(0.04)
*R* ^2^	0.19	0.34	0.23	0.40	0.34	0.12	0.40	0.40	0.42
*F*	13.04^***^	24.00^***^	16.69^***^	30.75^***^	24.39^***^	7.67^***^	31.23^***^	27.03^***^	25.46^***^

*N* = 290 subordinates, 51 supervisors; ^**^p < 0.01, ^***^p < 0.001 (two-tailed); Unstandardized coefficients are presented. Standard errors are reported in parentheses.

We used Model 4 of the PROCESS macro in SPSS, recommended by Hayes (2013), to test the mediating role of positive mood in the relationship between leader humility and affective commitment. We calculated the bias-corrected bootstrap 95% confidence interval of the indirect effect with 10,000 repetitions. Results showed that leader humility was positively related to employee affective commitment through positive mood (*β* = 0.14, SE = 0.03, 95% CI [0.10, 0.20]), supporting Hypothesis 3. Similarly, we used Model 6 of the PROCESS macro in SPSS to test the sequential mediation. Results showed that leader humility was positively related to employee proactive behavior through positive mood and affective commitment (*β* = 0.07, SE = 0.02, 95% CI [0.04, 0.11]), supporting Hypothesis 5.

**Figure 5:**
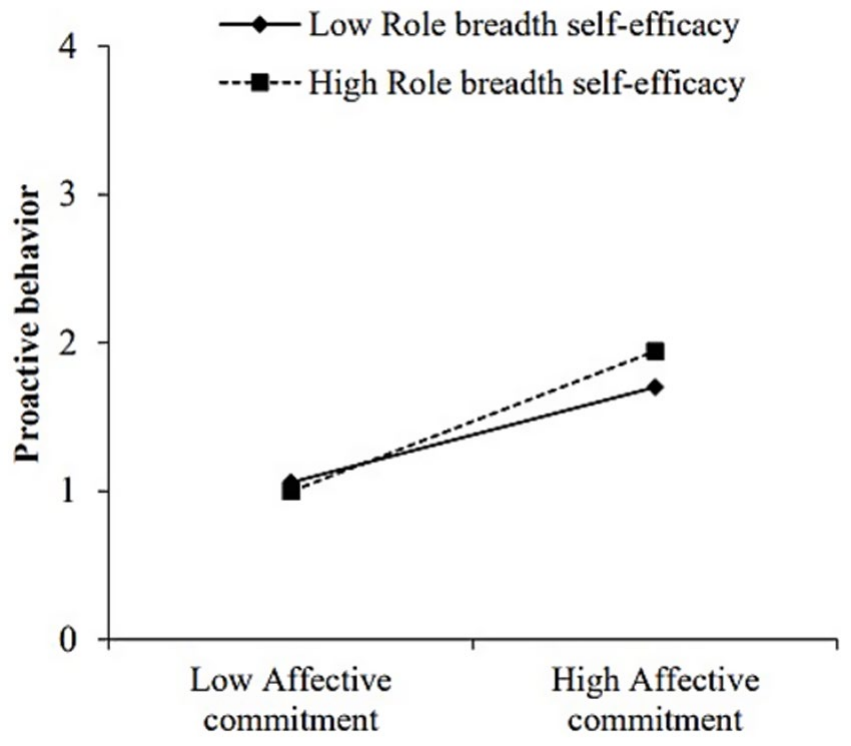
Moderating role of role breadth self-efficacy in the relationship between affective commitment and proactive behavior.

To test the moderating effects of role breadth self-efficacy, we examined the interactive effects of affective commitment and role breadth self-efficacy on proactive behavior using hierarchical regression analysis within SPSS. As recommended by Aiken and West (1991), we mean-centered affective commitment and role breadth self-efficacy before calculating their interaction term. As revealed by Model 8 in Table 3, role breadth self-efficacy was found to moderate the relationship between affective commitment and proactive behavior (*β* = 0.13, SE = 0.04, *p* < 0.05). To facilitate interpretation of the moderating effects, we plotted the interaction at 1 SD above and below the mean of role breadth self-efficacy and examined the simple slopes. As shown in Figure 5, the effect of affective commitment on proactive behavior was stronger at higher (+1 SD; *β* = 0.65, *p* < 0.001) than lower (−1 SD; *β* = 0.44, *p* < 0.001) levels of role breadth self-efficacy. These results supported Hypothesis 6.

Finally, we used the Mplus capabilities to test the moderated sequential mediation hypothesis. Results in Table 4 showed that role breadth self-efficacy moderated the indirect effects of leader humility on proactive behavior through positive mood and affective commitment. Specifically, the indirect effect of leader humility on proactive behavior was stronger at higher (+1 SD; *β* = 0.32, 95% CI [0.06, 0.63]) than at lower (**−**1 SD; *β* = 0.26, 95% CI [0.04, 0.51]) levels of role breadth self-efficacy. Thus, Hypothesis 7 received support.

**Table 4:** Conditional indirect effects for different values of role breadth self-efficacy for Study 2.

Moderator value	Indirect effect		
	estimate	SE	95%CI
Mean – 1SD	0.26	0.12	[0.04, 0.51]
Mean	0.29	0.13	[0.05, 0.57]
Mean + 1SD	0.32	0.15	[0.06, 0.63]

Indirect effect: leader humility → positive mood → affective commitment → proactive behavior; SE, standard error; SD, standard deviation; Bootstrap = 10,000 repetitions.

## Discussion

Drawing on AET, we used a scenario-based experiment study and a multi-source survey study to examine how and when leader humility relates to employee proactive behavior in China. Study 1 indicated that positive mood and affective commitment play a chain-mediating role in the relationship between leader humility and proactive behavior. Study 2’s multi-source data confirmed the sequential mediation, while demonstrating that role breadth self-efficacy strengthens both the affective commitment-proactivity link and the overall indirect effect.

These findings advance AET by delineating an affective process through which leadership events foster behavioral outcomes. While Study 1 provides causal evidence for the initial stages, we acknowledge that Study 2’s cross-sectional design limits definitive causal inferences about the full mediation sequence. Although common method bias was statistically ruled out and alternative models were rejected, we caution against interpreting the mediation and moderated mediation in Study 2 as conclusive evidence of causality. Future longitudinal designs tracking these mechanisms over time would strengthen causal claims.

### Theoretical implications

This study has several important theoretical implications. First, our findings reveal that positive mood and affective commitment operate as sequential mediators in the relationship between leader humility and employees’ proactive behaviors, particularly within Chinese organizational contexts. While researchers have emphasized the need to connect leadership with affect-related constructs (Scandura and Meuser, 2022; Tse et al., 2018), empirical research exploring the emotional mechanisms of leadership in Chinese workplaces remains limited (Wan et al., 2022). Grounded in AET, this study highlights how positive mood and affective commitment form a critical sequential chain translating leader humility into proactive actions. This not only validates AET’s utility in capturing emotion-driven dynamics but also underscores its cultural relevance in China, where affective bonds are central to explaining how leadership influences behaviors.

Second, the study expands the theoretical boundaries of leader humility research by introducing role breadth self-efficacy as a key individual-level moderator. Existing literature on humility’s contingencies has mostly emphasized the perspective of relationships between leader and member (e.g., Carnevale et al., 2019; Chen et al., 2018), social cues of leaders, such as perceived leader power and leader humility authenticity (e.g., Wang et al., 2018; Zhang and Liu, 2019), and employee values (e.g., Lin et al., 2019). In contrast, we draw on AET to show that role breadth self-efficacy moderates the effect of affective commitment on proactivity, and by extension, the indirect influence of leader humility on proactivity. This finding not only confirms AET’s relevance in explaining boundary conditions but also answers the recent call to explore individual differences in leader humility research (Kelemen et al., 2023).

### Practical implications

This study also offers important implications for practice. First, this study confirms that leader humility positively influences employee affective reactions and proactive behavior. To foster such behavior, organizations should develop human resource practices that cultivate leader humility. For instance, training programs can help supervisors recognize the value of humility and encourage its expression, such as acknowledging mistakes and highlighting subordinate contributions. Organizations may further develop supervisors’ growth mindset and relational identity, both empirically linked to leader humility (Wang et al., 2018). Additionally, selection processes for supervisory roles should incorporate assessments of candidates’ humility potential.

Second, this study found that positive mood and affective commitment are the processes that leader humility influences employee proactive behavior. Such findings suggest that organizations could benefit from enhancing these affective underpinnings. According to previous studies, a positive mood emerges when leaders display a positive mood and employees help others at work (Liu et al., 2017; Sonnentag and Grant, 2012). Affective commitment could be fostered by leaders providing social support or mentoring (Lapointe and Vandenberghe, 2017; Panaccio and Vandenberghe, 2009).

Third, this study demonstrates that leader humility more effectively promotes proactive behavior among employees with higher role breadth self-efficacy. As this efficacy reflects employees’ perceived capability to perform broader roles (Parker, 1998), organizations can amplify humility’s impact by developing this characteristic through structured interventions. For example, job enrichment programs such as gradual responsibility expansion via cross-functional project rotations build efficacy through structured mastery experiences (Beltrán-Martín et al., 2017). Organizations may also create practice environments featuring simulated low-stakes scenarios with mentor guidance, enabling safe initiative-taking skill development. Furthermore, integrating skill development recognition into reward systems reinforces efficacy growth beyond core task performance.

### Limitations and future research

Our study, however, has some limitations that need future research to address. First, while the experimental design offers stronger evidence for the causal direction of the proposed model, it relies on hypothetical scenarios with MBA students rather than capturing real-world behaviors of employees. This approach potentially limits generalizability. Future studies could test these hypotheses through field experiments in actual organizational settings.

Second, Study 2 collected multi-source data (supervisors and subordinates) to comprehensively examine the employee–supervisor relationship. However, data were collected at a single point in time, restricting the capacity to establish conclusive causal relationships. While our mixed-methods approach provides convergent support for the theoretical model, we recommend that future research adopt longitudinal designs to further validate these relationships.

Third, this study adopts the lens of AET to understand how leader humility affects employee proactive behavior in China. Future research could also benefit from investigating the mechanism from the relational perspective because relations (e.g., the relationship between leaders and employees) are also particularly important in Chinese culture (Hofstede et al., 2010). For example, leader humility may strengthen employees’ leader identification or perceived leader–member exchange, which in turn encourages employees to exhibit proactive behavior. Additionally, examining alternative mediating pathways such as cognitive trust or moral identity could provide deeper theoretical insights.

Fourth, according to AET, this study only examined the moderating role of role breadth self-efficacy, which captures employees’ self-perceived ability to act proactive behavior. Future studies can enrich the possible boundaries of how leader humility exerts influence on employee outcomes by exploring other individual differences. For example, for employees with higher levels of extraversion which reflects the extent to be susceptible to positive mood inductions (Larsen and Ketelaar, 1989), leader humility may elicit a more positive mood, and more affective commitment and proactive behavior could be spurred. Similarly, dispositional factors like regulatory focus and resilience, as well as situational perceptions such as psychological safety, might influence how employees translate affective commitment into sustained proactive efforts.

Finally, future research should explore how cultural differences might influence the relationships examined in this study. Given that cultural norms shape expectations regarding leadership styles and interpersonal behaviors (Lee et al., 2014), understanding potential variations across contexts could offer valuable insights for global organizations. Specifically, studies could investigate whether the observed effects are consistent across cultures or moderated by specific cultural factors.

## Conclusion

This study clarifies why and when leader humility drives employee proactive behavior by identifying a sequential affective pathway through which leader humility triggers positive mood, fosters affective commitment, and in turn spurs proactive behavior, while revealing role breadth self-efficacy as a critical boundary condition amplifying this process. Theoretically, we contribute to AET by demonstrating how leader humility operates as an affective event through sequential emotional mechanisms to shape proactive behavior. For leader humility research, we advance understanding by pinpointing not just that humility matters but how its influence unfolds through cumulative affective reactions. In proactivity literature, we uniquely show that role breadth self-efficacy specifically strengthens the link between affective commitment and action, a mechanism underemphasized in prior work. Methodologically, combining experimental rigor (Study 1) with multi-source field data (Study 2) provides robust validation for the proposed model. Limitations include Study 2’s cross-sectional design and cultural specificity, suggesting future longitudinal studies in non-Confucian settings. Practically, organizations can cultivate leader humility through approachability and appreciation training while enhancing role breadth self-efficacy via job enrichment, thereby leveraging these affective pathways to boost proactive behavior essential for organizational adaptability.

## Data Availability

The raw data supporting the conclusions of this article will be made available by the authors, without undue reservation.

## Appendix

**Table A1:** Experimental scenarios for Study 1.

Group	A: Leader humility group	B: Transactional leadership group
Experiment instructions	1. Before filling out the questionnaire, provide participants with a simulated scenario description: *“You currently work at a telecommunications equipment company. The company’s main business involves the R&D, production, and sales of mobile communication products. The company has been growing steadily over the past decade, and its market share has been increasing. You have worked at this company for over five years. Currently, you are a Customer Service Supervisor in the Customer Service Department. Your main responsibilities include handling customer orders and maintaining customer relationships.”* 2. Explain the corresponding leadership style. 3. Instruct participants to fill out the questionnaire based on the descriptions provided.	
Leader humility manipulation	“Dong Wang, a manager in the marketing department, is your immediate supervisor. He is a leader who treats others with respect and kindness. He is willing to listen and learn from followers, and acknowledge contributions from his/her followers when he/she succeeds. Also, he is self-aware and truthful about his competency and weakness, and enables followers to act and inspire them to do greater things, recognizing others for their accomplishments.”	“Dong Wang, a manager in the marketing department, is your immediate supervisor. He rewards employees who complete the assigned tasks, and punishes employees who do not meet job expectations. When there are small work problems, he does not make any concessions and lets subordinates solve them by themselves. But once the work problems become serious, he will take action to prevent bad performance.”
